# The Significance of Selected C-C Motif Chemokine Ligands in Colorectal Cancer Patients

**DOI:** 10.3390/jcm11071794

**Published:** 2022-03-24

**Authors:** Monika Zajkowska, Maciej Dulewicz, Agnieszka Kulczyńska-Przybik, Kamil Safiejko, Marcin Juchimiuk, Marzena Konopko, Leszek Kozłowski, Barbara Mroczko

**Affiliations:** 1 Department of Neurodegeneration Diagnostics, Medical University of Bialystok, 15-269 Bialystok, Poland; maciejdulewicz@gmail.com (M.D.); agnieszka.kulczynska-przybik@umb.edu.pl (A.K.-P.); barbara.mroczko@umb.edu.pl (B.M.); 2 Department of Oncological Surgery with Specialized Cancer Treatment Units, Maria Sklodowska-Curie Oncology Center, 15-027 Bialystok, Poland; kamil.safiejko@gmail.com (K.S.); jumedica.onkologia@gmail.com (M.J.); marzeniedoc@yahoo.com (M.K.); leszek@kozlowski.pl (L.K.); 3 Department of Biochemical Diagnostics, Medical University of Bialystok, 15-269 Bialystok, Poland

**Keywords:** CRC, CCL2, CCL4, CCL15, diagnostic utility

## Abstract

Colorectal cancer (CRC) is one of the most frequently diagnosed neoplasms. Despite the advances in diagnostic tools and treatments, the number of CRC cases is increasing. Therefore, it is vital to search for new parameters that could be useful in its diagnosis. Thus, we wanted to assess the usefulness of selected CC chemokines (CCL2, CCL4, and CCL15) in CRC. The study included 115 subjects (75 CRC patients and 40 healthy volunteers). The serum concentrations of all parameters were measured using a multiplexing method (Luminex). The CRP levels were determined by immunoturbidimetry, and the classical tumor markers (CEA and CA 19-9) were measured using CMIA (chemiluminescent microparticle immunoassay). The concentrations of all parameters were higher in the CRC group when compared to the healthy controls. The diagnostic sensitivity, specificity, positive and negative predictive value, and area under the ROC curve (AUC) of all estimated CC chemokines were higher than those of CA 19-9. Interestingly, the obtained results also suggest CCL2’s significance in the determination of local metastases and CCL4’s significance in the determination of distant metastases. However, further studies concerning the role of selected CC chemokines in the course of colorectal cancer are necessary to confirm and to fully clarify their diagnostic utility and their clinical application as markers of CRC development.

## 1. Introduction

Colorectal cancer (CRC) is one of the most frequent malignancies worldwide, being the second most common malignancy in men and third in women, and accounting for almost 11% and over 9% of all cancer cases, respectively. According to the World Health Organization (WHO), the global incidence of CRC is almost 2 million new cases per year, with approximately 920,000 deaths annually. Importantly, there is an observed increase in both the incidence and the mortality of colorectal cancer, as estimated year-to-year. It was predicted by WHO that the number of new CRC cases may exceed 3,000,000 in 2040, with the number of fatalities reaching 1.5 million per year [1,2]. What should be stressed is CRC is a preventable disease—even in up to 50% of cases—by some modifications of lifestyle, such as a high-fiber and a balanced diet, moderate physical activity, or avoidance of alcohol or smoking [3,4]. 

Diagnosis of colorectal cancer as early as possible, particularly in the asymptomatic stages, when the tumor is still non-malignant, and initiation of appropriate treatment is of principal importance for patients’ survival. The currently used methods of CRC detection include colonoscopy and sigmoidoscopy, as well as imaging diagnostics with the use of computed tomographic colonography and the magnetic resonance method. Although substantial progress has been made in this field in recent years, in the case of small lesions, with a mass not exceeding 1 g, these techniques may be ineffective. Another diagnostic tool useful in the detection of colorectal cancer are tumor markers, mainly glycoproteins or enzymes, which are synthesized by tumor cells. Tumor markers have a particular utility not only in detecting of malignancies and determining tumor advancement, but also in monitoring of treatment and early detection of recurrence [5,6]. The example of tumor markers employed in the diagnosis of CRC are CEA and CA 19-9. Unfortunately, the diagnostic usefulness of these biomarkers is relatively low and they are not specific to the colorectal cancer only [7]. Taking into account the above premises, there is an urgent need to find new diagnostic markers, the use of which will allow for detection of a developing cancer even earlier than before.

Increasing evidence suggests that small (8–12 kDa) inflammatory proteins known as chemokines are key regulators of angiogenesis, including pathological angiogenesis. They are a large family composed of 50 members. The main role of these cytokines is to direct the recruitment and the relocation of cells to locations of inflammation or injury. They are divided into four classes according to the location and the number of cysteine residues at the amino terminus. The CC-chemokine group has two adjacent cysteine residues, and the CXC chemokine group has two cysteine residues detached by an amino acid. The chemokine CX3C group has 3 amino acids between 2 cysteine residues and the C-chemokine group has only1 cysteine residue at the amino terminus. Of the four chemokine groups, the largest group is the CC chemokine group which includes a total of 28 members across all species. This is followed by the chemokine CXC group (17 members), with chemokines CX3C and XC having 1 and 2 members, respectively. All of these proteins exert their biological properties by interacting with G-protein-coupled transmembrane chemokine receptors found on the cell membrane of specific effector cells. The nomenclature of chemokines and their receptors results directly from their classification. At present, there are 19 receptors corresponding to specific groups of chemokines. Despite their large number, these receptors are structurally similar and they are activated in an analogous manner to the chemokines themselves [8].

The current state of knowledge allows us to suspect that chemokines and their receptors play a significant role in cancer development [9]. In tumor growth and metastasis, chemokines and their receptors exert a multifaceted effect on regulating angiogenesis, tumor cell proliferation, and apoptosis, mediating tumor cell metastasis in an organ-specific manner [10]. It is postulated that the CC and CXC chemokines could be the most active in the regulation of angiogenesis [11,12]. That is why the aim of our study was an attempt to clarify and to assess the usefulness of selected CC-chemokine measurement (CCL2, CCL4 and CCL15) in patients with colorectal cancer compared to the healthy volunteer group. We have also compared the obtained results to comparative, routinely used tumor markers (CA 19-9, CEA) and CRP (C-reactive protein), which is an inflammatory parameter. 

## 2. Materials and Methods

### 2.1. Patients

The study included 75 colorectal cancer (CRC) patients diagnosed by the oncology group (Table 1). The patients were treated in the Department of Oncological Surgery with Specialized Cancer Treatment Units, Maria Sklodowska-Curie Oncology Center, Bialystok, Poland. Tumor classification and staging were conducted in agreement with the International Union Against Cancer Tumor–Node–Metastasis (UICC-TNM) classification. The histopathology of colorectal cancer was based on the examination of tissue samples with the use of a microscope. Moreover, all patients were grouped according to not only tumor stage (TNM), but also depth of tumor invasion (T factor), the presence of lymph node (N factor), and distant metastases (M factor), as well as the histological grade (G factor) of the tumor. The pretreatment staging procedures included physical and blood examinations, computed tomography (CT) and—in case of patients with rectal cancer—magnetic resonance imaging (MRI) of the small pelvis. Additionally, all patients were assessed according to the Eastern Cooperative Oncology Group (ECOG) score. The control group comprised 40 healthy volunteers. For each patient qualified for the control group, the following exclusion criteria was applied: active infections and symptoms of an infection (both bacterial and viral); other comorbidities which can affect cytokine concentrations (respiratory diseases, digestive tract diseases); or systemic diseases such as lupus, rheumatoid arthritis, or collagenosis.

### 2.2. Biochemical Analyses

Venous blood samples were collected from each patient into a tube with a clot activator (S-Monovette, SARSTEDT, Numbrecht, Germany), centrifuged to obtain serum samples, and stored at −80 °C until assayed. The tested chemokines were measured with the use of a Luminex 200 analyzer (Thermo Fisher Scientific, Waltham, MA, USA) (multiplexing, multiparametric, fluorescence laser reading system on microspheres for the simultaneous determination of multiple parameters) and Luminex Human Discovery assay plates, provided by R&D systems, Abingdon, UK. According to the manufacturer’s protocols, duplicate samples were assessed for each standard, control, and sample. The serum levels of classical tumor markers were measured with chemiluminescent microparticle immunoassay (CMIA) (Abbott, Chicago, IL, USA); and, for the analysis of the CRP concentration, the immunoturbidymetric method (Abbott, Chicago, IL, USA) was used according to the manufacturer’s protocols.

### 2.3. Statistical Analysis

Statistical analysis was performed by RStudio. The introductory statistical analysis (using the Shapiro–Wilk test) exposed that the tested parameters and tumor marker levels did not follow normal distribution. Therefore, statistical analysis between the groups was performed with the use of the U Mann–Whitney test, the Kruskal–Wallis test, and a multivariate analysis of various data by the post-hoc Dwass–Steele–Critchlow–Flinger test. The data were presented as a median and a range. Diagnostic sensitivity, specificity, and the predictive values of positive and negative test results (SE, SP, PPV, and NPV, respectively) were calculated by using the cut-off values which were calculated by the Youden’s index (as a criterion for selecting the optimum cut-off point) and for each of the tested parameters they were as follows: CCL2—426.13 pg/mL, CCL4—274.45 pg/mL, CCL15—2607.49 pg/mL, CA 19-9—5.30 U/mL, CEA—1.70 ng/mL, and CRP—2.50 mg/L. We also defined the receiver operating characteristics (ROC) curve for all of the tested parameters, tumor markers, and for the CRP to estimate diagnostic accuracy, and we performed a Spearman’s rank correlation test. Statistically significant differences were defined as comparisons resulting in *p* < 0.05. 

## 3. Results

Table 2 shows the serum levels of the CCL2, CCL4, CCL15, CA 19-9, CEA, and CRP in patients with colorectal cancer and in the control group. After performing the non-parametric U Mann–Whitney test comparing the concentrations obtained in both groups, we observed that the levels of CCL2, CCL4, CEA, and CRP in the entire cancer group were significantly higher (in all cases *p* < 0.05).

In addition, we performed a more thorough analysis with use of Kruskal–Wallis and Dwass–Steel–Critchlow–Fligner tests after the division of the total CRC group into advancement groups (TNM I-IV). As a result of this analysis, we obtained significant results for almost all parameters (Table 3). Interpreting the obtained results, it can be suggested that the concentration of CCL4, CEA, and CA 19-9 increases significantly with the advancement of neoplastic changes, and it may be related not only to the number of neoplastic cells, but also to their spread—as TNM stage III is associated with the presence of metastases to nearby lymph nodes and stage IV with distant metastases. Interestingly, the CRP analysis confirms the inflammatory theory of neoplasm, as statistically significant differences were obtained only in the case of comparisons between the control group and individual stages of cancer.

Considering the fact that in the subgroups of TNM stages I and II, the number of patients did not exceed 20, which may affect the accuracy of the obtained results, we decided to confirm them using the U Mann–Whitney test. We divided the group of all CRC patients into the group of less-advanced neoplasms (TNM I + II) and the group of advanced neoplasms (TNM III + IV). In addition, we divided the group of advanced neoplasms into separate TNM stages (III and IV) due to the sufficient number of patients in each subgroup to perform a precise analysis in different subgroups and in comparison to the control group. The results obtained were similar to those in previous analyses. Interestingly, we observed significant differences between controls and III stage TNM in the case of CCL2, which may suggest its participation in local lymph node metastasis processes. In the case of CCL4, we observed differences between the control and the most advanced stage of CRC, and what is of utmost importance, significant differences between all advancement CRC stages (similarly to CA 19-9). In comparison between the control group and all advancement stages, CEA and CRP revealed significance; but, in case of differences between TNM stages, significant results were obtained only in case of CEA between less-advanced stages and distant stage metastases (Table 4). 

Table 5 shows the sensitivity, specificity, positive and negative predictive values (SE; SP; PPV; NPV, respectively), and the relationship between them with the use of the area under the ROC curve (AUC) of all newly tested parameters. We indicated that the highest SE from all parameters revealed CCL4 (76%). The observed value is higher than SE of commonly used tumor markers such as CEA (75%), CA 19-9 (51%) and C-reactive protein (73%). In the case of SP, the highest value was observed for CCL2 (60%) and it was higher than SP of CA 19-9 (48%), but the highest specificity from all parameters was observed in case of CRP (78%) and CEA (70%). Positive and negative predictive values were highest in case of CCL2 and CCL4 (72%/47% and 74%/53%, respectively). These values were slightly lower than PPV and NPV of CEA and CRP. What is more, the SE, SP, PPV, and NPV values of all newly tested parameters (CCL2, CCL4, CCL15) were higher than CA 19-9, which confirms their higher usefulness in case of patients with CRC than the routinely used marker.

**Figure 1:**
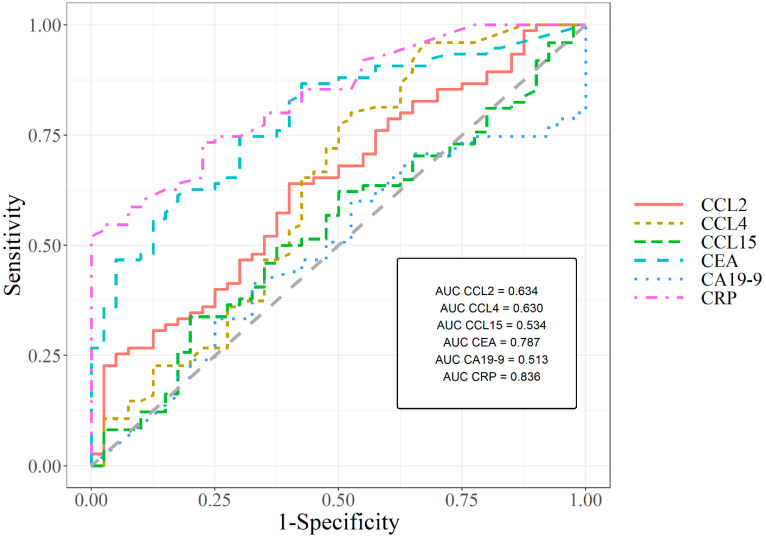
Receiver operating characteristics for all significant ROC analysis results.

We noticed that the CCL2 and CCL4 areas under the ROC curve (0.634; 0.630, respectively) in the entire group of colorectal cancer were highest from all newly tested parameters, but lower than AUC for CEA and CRP. Additionally, similar to previously mentioned statistical parameters, in the case of all tested CC chemokines, AUC was higher than AUC for CA 19-9. A graphical version of all of the significant ROC analysis results is shown in Figure 1. The AUCs for the tested parameters, as for generally used tumor markers and combined analysis, were significantly larger in comparison to AUC = 0.5 (borderline of the diagnostic usefulness of the test) (*p* < 0.05 in all cases).

In order to complete the statistical analysis, we checked the Spearman’s rank correlation coefficient to measure and to show the strength and the direction of monotonic association between variables in the CRC group. Obtained results are shown in Table 6. We observed a strong positive correlation for one of the tested parameters (CCL4) and the tumor TNM stage. This may confirm that the increasing concentration of this parameter is related to the number of neoplastic cells. This fact was also observed during the Kruskal–Wallis and Dwass–Steel–Critchlow–Fligner tests. In the case of the remaining parameters (CEA and CA 19-9), we also observed a similar correlation but of moderate strength. Moderate, positive correlation was also observed between the CEA and the CCL4 concentrations, and concentrations of both markers (CEA and CA 19-9). The rest of the observed correlations revealed weak strength (coefficient < 0.40). Interestingly, we observed also one negative but weak correlation between the CCL2 and the CCL15 concentrations.

## 4. Discussion

At present, in case of patients with colorectal cancer it is clinically important to search for new prognostic or predictive markers, as they might influence postoperative decisions. Generally, the guidelines for CRC are mainly based on the basis of, e.g., the TNM stage or the molecular characteristics of the tumor. In some cases, the decision whether to use or not to use adjuvant chemotherapy requires additional tests such as for a serum CEA level or an expression of p53/Ki67 [13]. In accordance with that, researchers are searching for different, new parameters to find markers for the highly accurate and non-invasive tests for colorectal cancer.

We indicated that the serum concentration for CCL2 was statistically higher in the group of colorectal cancer patients when compared to healthy controls (*p* = 0.02). Similar results were obtained in the work of De la Fuente López et al. [14]. These authors revealed that not only plasma levels, but also the concentration of this parameter in CRC tissue lysates is significantly higher when compared to healthy mucosa. Nevertheless, as the number of samples in these investigations were low (25 tissues; 32 CRC patients and 15 healthy patient plasma samples), these results needed a verification. Another work confirmed that a higher expression of CCL2 can be found in cancer tissue, and it is connected with a negative prognosis in CRC patients [15]. Interestingly, some researchers have indicated that an overexpression of CCL2 is associated with increased metastatic potential [16]. A different study by Nardelli et al. [17] also established that circulating CCL2 levels were associated with the presence of CRC, but the number of patients in this study was also insufficient (20 CRC patients and 20 healthy volunteers). A different research group also suggested that higher CCL2 levels may be considered as a prognostic factor in CRC, but this study was performed with the use of serum from 45 patients, and peculiarly, the control group was not included [13]. Therefore, our research carried out on a much larger group of patients with the use of a sufficiently large group of healthy volunteers, finally confirms the previously mentioned fragmentary reports. 

Some studies also assessed the changes of CCL2 concentration after surgery or perioperatively. Hua et al. [18] discovered that elevated levels of this parameter are associated with a high risk of overall mortality. On the contrary, a study by Watanabe et al. [19] revealed that a decrease in the CCL2 ratio between tumoral and normal adjacent tissues is associated with lymph node involvement, and it could predict a poor prognosis. This discrepancy may be related not only to the difference in the material used for research, but also to the calculated ratio. Its reduction may be caused not only by lower expression in neoplastic tissues, but also by increased expression in healthy tissues. Interestingly, when analyzing the concentration of CCL2 in the control group and the study group by TNM stage, we showed a significant relationship between stage III and the control group, which may be a confirmation of the Watanabe et al. [19] investigation, which pointed out the relationship between CCL2 and the appearance of local lymph node metastases. In addition, the work of Johdi et al. [20] showed that there were no differences in the serum concentration of CCL2 and CRC, polyp, and healthy subjects. However, these results were performed on a basis of only 20 samples from each group. In the work of Tonouchi et al. [21], CCL2 levels were significantly raised 1 h after surgery, which suggests that this parameter can have a different role than as a marker of surgical insults, especially, as these differences did not correlate with IL-6 changes. However, a few days after surgery, the levels of this parameter were comparable to those before surgery. Due to discrepancies in the previously obtained studies, all previously mentioned results require further confirmation, which indicates a further plan for the continuation of our research.

We also found studies concerning the CCL2 concentration and expression in murine models [22,23]. These authors concluded that this cytokine may activate macrophages to become tumoricidal, resulting in the suppression of metastasis; and, they could be useful as biomarkers of colon cancer progression, which fully coincides with our discoveries.

On the other hand, one of the publications indicated that CCL2 did not show any differences between the adenoma group compared to the control group. This inconsistency may be related to a too-early period of changes leading to cancer progression. However, the serum samples used in the study were stored for many years and they were transported several times, which may have a significant impact on the results obtained by those researchers [24]. These results also suggest the need for further confirmation.

In the work of De la Fuente López et al. [14], CCL4 concentration was also found (similar to our study) to be significantly increased in CRC patients when compared to healthy controls. The previously mentioned work by Johdi et al. [20] also included the CCL4 determinations. Interestingly, in case of this parameter, significantly higher concentrations in the blood serum of patients with CRC compared to the control group were observed, as well as in the serum of patients with colorectal polyps. 

Remarkably, in the work of Krzystek-Korpacka et al. [25], it was shown that CCL4 concentrations in the case of CRC are significantly higher when compared to the control group. However, after division by tumor location (rectum and colon), it turned out that in the case of rectal cancer, these concentrations are the highest and the difference is statistically significant. This can be important information when attempting to personalize therapy, and it is indicative of the heterogeneity of CRC. Surprisingly, in the work of Pervaiz et al. [26], completely contradictory results were presented. In IV stage of the tumor’s advancement, statistically lower concentrations of CCL4 were demonstrated compared to the control group. These differences may be related to the number of patients, as the studies of Pervaiz et al. [26] were carried out on a group of 24 patients diagnosed with CRC, of which only 3 were at stage IV of CRC advancement. 

In the case of CCL15, for which we did not observe any statistical differences, we found only one study that assessed the concentration of this parameter in the course of CRC. Inamoto et al. [27] showed that the concentrations of CCL15 in patients with CRC are statistically significantly higher than in healthy subjects, not only in the entire study group, but also at various stages of CRC advancement. These differences are extremely difficult to explain, as both experiments involved a properly large group of patients. However, the results obtained by Inamoto et al. [27] were several times higher (median for the control group 9.4 ng/mL; for the tested group 17.8 ng/mL). Possibly these differences could be influenced by the method of determination (ELISA vs. Luminex) or the ethnicity of the patients (Asian vs. European).

Unfortunately, we have not found any other papers that would focus on demonstrating the dependence and statistical significance based on the division of the study group into stages of advancement. Therefore, we believe that our work is innovative in this matter, which significantly increases its value. A more accurate demonstration of the relationships between the control group and the study group may significantly affect the understanding of changes in the course of CRC. Interestingly, our results showed a significant relationship between CCL2 and III TNM stage of CRC, which may be associated with the formation of local lymph nodes metastases, and significant differences between the concentration of CCL4 in stage IV of CRC and the control group, which may indicate its involvement in the development of distant metastasis. Due to the fact that these are the first reports on these dependencies, it is advisable to confirm them in further analyses. 

According to our knowledge, the present study is also the first that assesses diagnostic criteria such as SE, SP, PPV, NPV, and ROC. However, parameters such as CCL2 and CCL4 showed high values (especially diagnostic sensitivity) compared to markers routinely used in diagnostics, and even higher than CA 19-9. This is significant evidence that these cytokines can contribute to the development of diagnostics and constitute an additional diagnostic parameter, e.g., in the case of detecting local and distant metastases. Perhaps a simultaneous analysis of the classical tumor markers and the tested cytokines would increase their diagnostic utility, which is an important task for the continuation of our research in the future. The only work assessing merely the SE and the SP of the CCL15 chemokine was the previously mentioned work of Inamoto et al. [27], whose results were significantly higher than ours (78.8%; 70% vs. 57%; 53%, respectively). These discrepancies may be due to the same reasons as for the concentrations of CCL15 described above.

We also tried to determine the correlations between the examined parameters, which showed that the concentration of CCL2 positively correlates with the concentration of CCL4 and negatively correlates with the concentration of CCL15. In contrast, CCL4 positively correlated with routine markers (CEA, CA 19-9), CRP protein, age (similar to CEA), and tumor stage (similar to CEA and CA 19-9). The study by De la Fuente López et al. [14] showed a significant correlation between CCL4 and the CD163 marker on pro-tumor macrophages and inflammatory mediators (VEGF, TNF-α). This indicates the high potential of CCL4 to induce infiltration of tumor-associated macrophages which may be related to tumor progression or metastases associated with high levels of CCL4, which was found in our study.

## 5. Conclusions

According to our knowledge, the current study is the first that links the diagnostic characteristics of CCL2, CCL4, and CCL15 with the well-established, classical tumor markers (CEA and CA 19-9) and CRP—which is the marker of inflammation—in CRC patients, and not only in the entire study group, but also in subjects divided according to TNM stage. The results obtained suggest the significant importance of CCL2 in the determination of local metastases and CCL4 in the case of distant metastases. However, after a careful analysis of our results and the results of other authors, it is certain that further studies concerning the concentrations of selected CC chemokines in the course of colorectal cancer are necessary to confirm and to clarify their diagnostic utility and their clinical application as potential non-invasive markers of CRC development. 

## Figures and Tables

**Table 1:** Characteristics of colorectal cancer and healthy patient groups.

Study Group		No. of Patients
Colorectal Cancer		75
	Gender:	
	Female	26
	Male	49
	Type:	
	Colon Cancer	25
	Rectal Cancer	41
	Sigmoid Cancer	9
	TNM Stage:	
	0	1
	I	15
	II	13
	III	25
	IV	21
	Depth of tumor invasion:	
	In situ	1
	T1	2
	T2	19
	T3	41
	T4	12
	Nodal involvement:	
	N0	34
	N1	25
	N2	16
	Distant metastasis:	
	M0	54
	M1	21
	Age:	33–89
Control Group		40
	Gender:	
	Female	12
	Male	28
	Age:	34–80

**Table 2:** Serum levels of tested parameters in cancer and control groups.

Parameter		Colorectal Cancer	Control Group	*p* *
CCL2 [pg/mL]	Me Min–Max	485.68 181.12–2033.23	371.81 72.80–1117.74	**0.02**
CCL4 [pg/mL]	Me Min–Max	378.81 142.60–655.50	272.03 83.58–933.72	**0.02**
CCL15 [pg/mL]	Me Min–Max	2853.88 204.17–12,750.00	2547.22 132.63–14,677.81	0.55
CA 19-9 [U/mL]	Me Min–Max	5.30 2.06–8199.90	5.39 2.06–33.34	0.82
CEA [ng/mL]	Me Min–Max	3.87 0.50–3688.00	1.02 0.50–15.64	**<0.001**
CRP [mg/L]	Me Min–Max	6.00 1.00–248.50	1.36 0.20–5.80	**<0.001**

* U Mann–Whitney test; CCL—chemoattractant cytokine ligand; CA 19-9—cancer antigen 19-9; CEA—carcinoembryonic antigen; CRP—C-reactive protein. The statistically significant results are presented in bold.

**Table 3:** Kruskal–Wallis and Dwass–Steel–Critchlow–Fligner tests analysis results.

Parameter		CCL2	CCL4	CCL15	CA 19-9	CEA	CRP
Kruskal–Wallis *p*-value		**0.05**	**<0.001**	0.22	**0.003**	**<0.001**	**<0.001**
Dwass–Steel–Critchlow–Fligner *p*-value	Control vs. I	0.35	0.99	0.70	0.19	0.34	**<0.001**
	Control vs. II	1.00	0.98	1.00	0.83	0.08	**<0.001**
	Control vs. III	**0.05**	0.58	0.99	0.50	**0.002**	**<0.001**
	Control vs. IV	0.52	**<0.001**	0.41	0.25	**<0.001**	**<0.001**
	I vs. II	0.54	0.92	0.65	0.98	1.00	0.99
	I vs. III	0.99	0.53	0.63	**0.033**	0.77	1.00
	I vs. IV	0.99	**<0.001**	0.99	**0.013**	**<0.001**	0.99
	II vs. III	0.31	0.19	0.99	0.17	0.68	0.95
	II vs. IV	0.93	**<0.001**	0.67	0.15	**<0.001**	0.99
	III vs. IV	0.91	**<0.001**	0.28	0.99	**0.005**	0.97

The statistically significant results are presented in bold.

**Table 4:** U Mann–Whitney test analysis results between control group and TNM subgroups.

Parameter		CCL2	CCL4	CCL15	CA 19-9	CEA	CRP
U Mann–Whitney test *p*-value	Control vs. I + II	0.44	0.99	0.91	**0.05**	**0.04**	**<0.001**
	Control vs. III + IV	**0.02**	**0.002**	0.82	0.10	**<0.001**	**<0.001**
	Control vs. III	**0.02**	0.32	0.87	0.30	**<0.001**	**<0.001**
	Control vs. IV	0.27	**<0.001**	0.24	0.14	**<0.001**	**<0.001**
	I + II vs. III + IV	0.32	**<0.001**	0.89	**<0.001**	**<0.001**	1.00
	I+II vs. III	0.17	**0.03**	0.39	**<0.001**	0.10	0.70
	I+II vs. IV	0.82	**<0.001**	0.48	**<0.001**	**<0.001**	0.67

The statistically significant results are presented in bold.

**Table 5:** Diagnostic criteria of tested parameters in patients with colorectal cancer.

Tested Parameters	Diagnostic Criteria	Colorectal Cancer
CCL2	SE	64%
	SP	60%
	PPV	75%
	NPV	47%
	AUC	0.634
CCL4	SE	76%
	SP	50%
	PPV	74%
	NPV	53%
	AUC	0.630
CCL15	SE	57%
	SP	53%
	PPV	69%
	NPV	40%
	AUC	0.534
CA 19-9	SE	51%
	SP	48%
	PPV	64%
	NPV	34%
	AUC	0.513
CEA	SE	75%
	SP	70%
	PPV	82%
	NPV	60%
	AUC	0.787
CRP	SE	73%
	SP	78%
	PPV	86%
	NPV	61%
	AUC	0.836

SE—sensitivity; SP—specificity; PPV—positive predictive value; NPV—negative predictive value.

**Table 6:** Spearman’s rank correlation coefficient for tested variables.

Tested Variables	CCL2	CCL4	CCL15	CA 19-9	CEA	CRP	Age
CCL4	**0.34** ***p* < 0.001**	-					
CCL15	**−0.24** ***p* = 0.04**	−0.06 *p* = 0.60	-				
CA 19-9	−0.01 *p* = 0.96	**0.39** ***p* < 0.001**	−0.04 *p* = 0.73	-			
CEA	0.07 *p* = 0.56	**0.56** ***p* < 0.001**	0.09 *p* = 0.46	**0.51** ***p* < 0.001**	-		
CRP	0.18 *p* = 0.13	**0.32** ***p* = 0.01**	0.04 *p* = 0.74	−0.01 *p* = 0.90	0.17 *p* = 0.16	-	
Age	0.18 *p* = 0.11	**0.39** ***p* < 0.001**	−0.00 *p* = 0.98	0.18 *p* = 0.13	**0.36** ***p* < 0.001**	−0.07 *p* = 0.55	-
TNM stage	0.02 *p* = 0.84	**0.62** ***p* < 0.001**	0.06 *p* = 0.60	**0.43** ***p* < 0.001**	**0.57** ***p* < 0.001**	0.05 *p* = 0.67	**0.34** ***p* < 0.001**

The statistically significant results are presented in bold.

## Data Availability

The data presented in this study are available on request from the corresponding author. Key data are stated in the text.
